# Role of apoptosis in the pathogenesis of COPD and pulmonary emphysema

**DOI:** 10.1186/1465-9921-7-53

**Published:** 2006-03-30

**Authors:** Ingel K Demedts, Tine Demoor, Ken R Bracke, Guy F Joos, Guy G Brusselle

**Affiliations:** 1 Department of Respiratory Diseases, Ghent University Hospital, Belgium

## Abstract

Chronic obstructive pulmonary disease (COPD) is characterised by chronic inflammation of the airways and progressive destruction of lung parenchyma, a process that in most cases is initiated by cigarette smoking. Several mechanisms are involved in the development of the disease: influx of inflammatory cells into the lung (leading to chronic inflammation of the airways), imbalance between proteolytic and anti-proteolytic activity (resulting in the destruction of healthy lung tissue) and oxidative stress. Recently, an increasing number of data suggest a fourth important mechanism involved in the development of COPD: apoptosis of structural cells in the lung might possibly be an important upstream event in the pathogenesis of COPD. There is an increase in apoptotic alveolar epithelial and endothelial cells in the lungs of COPD patients. Since this is not counterbalanced by an increase in proliferation of these structural cells, the net result is destruction of lung tissue and the development of emphysema. Data from animal models suggest a role for Vascular Endothelial Growth Factor (VEGF) in the induction of apoptosis of structural cells in the lung. Other mediators of apoptosis, such as caspase-3 and ceramide, could be interesting targets to prevent apoptosis and the development of emphysema.

In this review, recent data on the role of apoptosis in COPD from both animal models as well as from studies on human subjects will be discussed. The aim is to provide an up to date summary on the increasing knowledge on the role of apoptosis in COPD and pulmonary emphysema.

## Review

Chronic Obstructive Pulmonary Disease (COPD) is a chronic respiratory disease that is associated with an abnormal inflammatory response of the lungs to noxious particles or gases (mainly cigarette smoke). This leads to chronic bronchitis-bronchiolitis (small airways disease) and/or emphysema that cause airflow limitation that is not fully reversible. [1]. COPD is the fifth leading cause of death worldwide, accounting for more than 2 500 000 deaths every year (WHO world health report 2002). Moreover, the prevalence and mortality of COPD are expected to increase in the coming decades [2].

Several mechanisms contribute to the pathogenesis of COPD [3]. First, the inhalation of noxious particles such as cigarette smoke causes the influx of inflammatory cells into the airways and lungs, leading to chronic inflammation. Different kinds of inflammatory cells (macrophages, neutrophils, CD8+ T lymphocytes) have been described to participate in the inflammatory response in the airways of COPD patients.

Second, there is a disruption of the balance between proteolytic and anti-proteolytic molecules in the lungs of COPD patients, resulting in an increased proteolytic activity [4]. This causes the destruction of healthy lung parenchyma, which leads to the development of emphysema. This increase in proteolytic activity may be a consequence of inflammation (release of proteolytic enzymes by inflammatory cells such as macrophages and neutrophils) or may arise from genetic factors (eg alpha-1 antitrypsin deficiency).

A third mechanism involved in the pathogenesis of COPD is oxidative stress, which occurs when reactive oxygen species are produced in excess of the antioxidant defence mechanisms [3]. Oxidants are generated in the airways by cigarette smoking or are released from inflammatory leukocytes and epithelial cells. Oxidative stress can lead to cell dysfunction or cell death and can induce damage to the lung extracellular matrix. Moreover, oxidative stress influences the proteinase-antiproteinase imbalance by activating proteases and inactivating antiproteinases. Additionally, oxidants contribute to the inflammatory reaction by activating the transcription factor NF-κB and thus inducing the transcription of pro-inflammatory genes. In conclusion, it is clear that these three processes (chronic inflammation, proteinase/anti-proteinase imbalance and oxidative stress) involved in the pathogenesis of COPD are not independent mechanisms and several interactions between these processes occur during the development of the disease.

**Figure 1:**
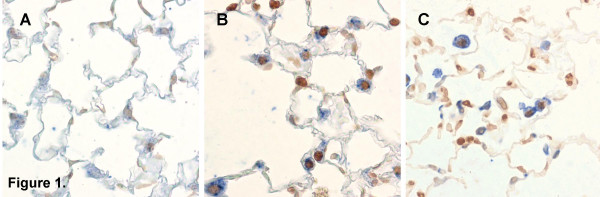
**Identification of apoptotic cells in the lung in an animal model of COPD**. TUNEL-staining demonstrating an increase in apoptotic cells (dark brown nuclei) in the lungs of mice exposed to cigarette smoke (Figure 1B-C) compared to air-exposed animals (Figure 1A). Double stainings against pro-surfactant-protein C (Figure 1B, alveolar epithelial cells identified as blue pro-surfactant-protein C+ cells) or CD45 (Figure 1C, inflammatory cells identified as blue CD45+ cells) and TUNEL-staining (Figure 1B-C, dark brown nuclei) demonstrating apoptosis of both structural and inflammatory cells (own unpublished data).

Recent data from both animal models of COPD (Figure 1, own unpublished observations) as well as from studies in human subjects suggest that a fourth mechanism might be involved in the pathogenesis of COPD: disruption of the balance between apoptosis and replenishment of structural cells in the lung might contribute to the destruction of lung tissue in response to cigarette smoke, leading to emphysema.

### Apoptosis

**Figure 2:**
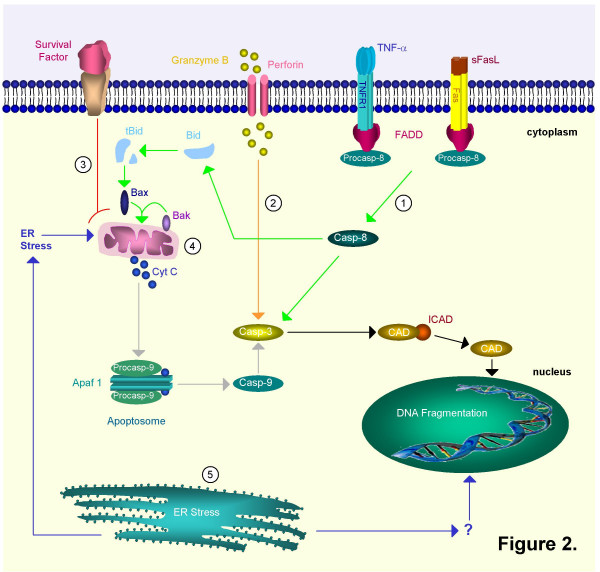
**Schematic representation of different pathways involved in apoptosis**. **Extrinsic pathway: 1. ****Ligand-death-receptor pathway (green): **death factors such as Fas ligand (FasL) and tumour necrosis factor (TNF) trigger apoptosis by binding on 'death receptors' such as Fas and Tumour Necrosis Factor Receptor 1 (TNFR1). FasL may be solubilized to sFasL by matrix metalloproteinases (MMP's). The death receptors recruit procaspase-8 by means of an adaptor protein, Fas associated death domain protein (FADD). After cleavage the mature caspase-8 then directly activates caspase-3 or cleaves Bid. Truncated Bid (tBid) interacts with Bax and Bak. A pore is formed in the outer mitochondrial membrane through which cytochrome c (Cyt C) is released. **2. ****Cytolytic effector cell pathway (orange): **cytotoxic T cells can release granzyme B and perforin, a pore-forming protein. Granzyme B activates caspase-3 through cleavage. It can also cleave caspase-8. **3. ****Growth factor depletion pathway (red): **deprivation of survival factors triggers Cyt C release through activation of Bax and Bak. **Intrinsic pathway: 4. ****Mitochondrial pathway (grey): **mitochondria release cytochrome c (Cyt C) in response to stress. Together with apoptotic protease activating factor-1 (Apaf-1) and procaspase-9, Cyt C will form the apoptosome complex. This results in the proteolytic activation of the procaspase. Mature caspase-9 can then proteolytically activate caspase-3 and other executioner caspases. **5. ****Endoplasmatic reticulum pathway (blue): **the ER can also induce apoptosis as a reaction to stress. It might do so by stimulating the mitochondrial pathway or by directly targeting the nucleus. In mice both caspase-7 and -12 are linked to this pathway. These different initiation pathways converge further downstream into activation of caspase-3. The effector caspase-3 cleaves ICAD (inhibitor of CAD) and releases it from CAD (caspase-activated DNAase). CAD translocates from the cytoplasm to the nucleus and can now act as active endonuclease and fragment DNA.

Apoptosis is a tightly regulated mechanism of cell death. This programmed cell death allows the elimination of unwanted, damaged or infected cells. At present, three different pathways that are involved in the regulation of apoptosis have been described (Figure 2). Different caspases (these are proteases with an important function in the regulation of apoptosis) are involved in these different pathways [5]. A first pathway is activated in response to extracellular signals and is mediated by binding of members of the tumour necrosis factor family (e.g. Fas ligand, TNF-α) to death receptors on the cell surface (e.g. Fas, TNFR). This results in the multimerization of the death receptor and the formation of the death inducing signalling complex (DISC), containing multiple adaptor molecules such as the Fas associated death domain (FADD). This FADD interacts with caspase-8. [6,7], leading to the autolytic activation from pro-caspase-8 to caspase-8. Caspase-8 then activates caspase-3 [8,9], which finally executes apoptosis by releasing caspase-activated DNAse (CAD) from its inhibitor (ICAD) with DNA fragmentation as a consequence [10]. This pathway is called the receptor mediated extrinsic pathway (Figure 2). Importantly, caspase-8 can also cleave the pro-apoptotic Bid [11], which then, through interaction with Bax and Bak, translocates to the mitochondria and causes the release of cytochrome C (see below). [12].

A second pathway, the mitochondrial intrinsic pathway, responds to physical and chemical stress signals by the release of cytochrome C from mitochondria. As a consequence, cytochrome C, apoptotic protease activating factor-1 (Apaf-1) and caspase-9 form the apoptosome. [13-15], resulting in the activation of caspase-9 which then activates caspase-3 and initiates the execution of apoptosis [16].

Finally, in the endoplasmatic reticulum pathway, caspase-12 is activated in response to stress signals such as hypoxia. [17,18]. In addition to these caspase-dependent pathways, it has been shown that noncaspase proteases can process and activate caspases directly (eg activation of caspase-3 by granzyme B) [19]. Moreover, the deprivation of survival signals such as growth factors can also induce apoptosis by mitochondrial release of cytochrome C. [20,21].

While apoptosis is a tightly regulated active mechanism leading to programmed cell death, cell necrosis is a rather uncontrolled process, which is accidental and in which the cell has no active role [22]. As a consequence, apoptosis seems to be a process that can be modulated to maintain cell viability, while necrosis is difficult to prevent.

Apoptosis is critical for the maintenance of normal tissue homeostasis and is in equilibrium with proliferation and differentiation. There is increasing evidence that disturbance of the balance between apoptosis and proliferation in lung tissue contributes to the pathogenesis of COPD. A limited number of descriptive studies in human subjects suggest a possible role for apoptosis in COPD, while an increasing number of experimental studies in animal models of COPD provides more insight into the association between cigarette smoking, apoptosis and the development of emphysema.

### Studies on human subjects

#### Apoptosis in human lung: ex vivo/in vitro

Several groups studied the role of apoptosis in the pathogenesis of COPD in human subjects, mostly by using lung tissue sections from COPD patients and controls (Table 1). Segura-Valdez et al described an increase in endothelial cell apoptosis in lung tissue sections from COPD patients compared to controls. Although less frequently, apoptotic alveolar epithelial cells, interstitial cells and inflammatory cells (neutrophils and lymphocytes) were also described in the lungs of COPD patients, while this was not the case in control subjects [23].

**Table 1:** Overview of studies on apoptosis in human lung.

**Reference**	**Patients**	**COPD defined by**	**Controls**	**Increased Apoptosis in patients**	**Increased Proliferation in patients**
Segura-Valdez [23]	Chronic Bronchitis Emphysema	-Medical history -Pulmonary Function -Histology/CT	-Male individuals who died from other causes than lung diseases smoking status unknown	-endothelial cells -alveolar epithelial cells -interstitial cells -inflammatory cells	NA
Majo [27]	Emphysema	-Pulmonary Function -Histology	-Never smokers -Smokers without emphysema	-No difference between groups	NA
Kasahara [28]	Emphysema	-Pulmonary Function	Non smokers	-alveolar epithelial cells	NA
		-Histology/CT	-Smokers without airway obstruction	-endothelial cells	
Yokohori [25]	Emphysema	-Pulmonary Function -Histology	-Asymptomatic smokers -Asymptomatic nonsmokers	-alveolar epithelial cells type II	alveolar epithelial cells type II
Imai [24]	Emphysema	-Pulmonary Function -Histology	-accidental death victims unused donor lungs for LTX -Smoking status unknown	-alveolar epithelial cells -endothelial cells -mesenchymal cells	Increased (cell type not specified)
Hodge [26]	COPD	-Medical history -Pulmonary Function	-Never smokers	-airway epithelial cells (obtained by BAL) -BAL T-cells	NA

BAL = bronchoalveolar lavage; CT = computed tomography; LTX = lung transplantation; NA = not available

Imai and colleagues described an increase in apoptotic cells (alveolar epithelial cells, endothelial cells and mesenchymal cells) in emphysematous lung tissue, as well as an increase in the activated subunits of caspase-3 (an important caspase in the execution of downstream events in apoptosis). Moreover, expression of the pro-apoptotic proteins Bax and Bad was detected in emphysema patients, while this was not the case in controls. The anti-apoptotic protein Bcl-2 was not detected in either normal or emphysematous lung tissue. Interestingly, increased cell proliferation was found in emphysematous lungs [24].

Other groups described similar findings, with an increase in both apoptosis and proliferation of alveolar wall cells in patients with emphysema compared to smokers without COPD and non-smokers [25]. Hodge et al described an increase in apoptosis of alveolar epithelial cells and T-cells from bronchial brushings and bronchoalveolar lavage in COPD patients compared to non-smoking controls. [26]. This increase in apoptosis in COPD patients persisted despite smoking cessation.

Others, on the contrary, did not find a significant difference in apoptotic alveolar wall cells in the lungs from smokers without emphysema compared to smokers with emphysema. [27]. However, in this study, apoptosis in smokers showed a bilinear relationship with the amount smoked: the apoptotic index decreased in smokers without emphysema to a minimum at 40 pack year, then increasing sharply as the pack year increased in smokers with emphysema. Interesting findings were obtained by Kasahara et al. These authors demonstrated an increase in apoptotic epithelial and endothelial alveolar septal cells in emphysematous lungs compared to non-smokers, smokers and primary pulmonary hypertension patients. [28]. Moreover, expression of VEGF and VEGF R2 protein and mRNA was significantly reduced in emphysema. The authors hypothesized that this decrease of endothelial cell maintenance factors, leading to endothelial alveolar septal death, may be part of the pathogenesis of emphysema. Recent data from other groups support this finding, by demonstrating that VEGF levels in induced sputum from COPD patients decreased with severity of COPD. [29]. However, while VEGF signalling may be required for the maintenance of the alveolar structures, the 936 C/T polymorphism of the VEGF gene (associated with lower VEGF plasma levels) was not associated with the development of COPD [30].

Altogether, several studies in human COPD patients describe an increase in apoptosis, especially in structural cells in the lung (Table 1). However, some points should be taken into consideration when interpreting these data. First, not all studies have studied changes in lung cell proliferation in addition to apoptosis. As mentioned above, in physiologic circumstances, apoptosis is in balance with processes such as proliferation and differentiation. As a consequence, when studying the role of apoptosis in diseases such as COPD, it is recommendable to evaluate changes in proliferation as well. By doing so, this will allow to discriminate between a net increase in apoptosis (not counterbalanced by an increase in proliferation and leading to the loss of structural lung cells and tissue) and an equal increase in both apoptosis and proliferation (where the loss of structural cells by apoptosis is prevented by the regeneration of structural cells in the lung). By using this approach, Calabrese and colleagues recently demonstrated that there was a significant increase in apoptotic alveolar epithelial cells in end-stage emphysema (particularly in emphysema due to α1-antitrypsin deficiency), while there was no difference in proliferation of alveolar septal cells between emphysema patients and controls [31].

Second, in some of these studies, the control groups used consisted of non-smokers or of smokers with significantly less pack years of cigarette smoking compared to the COPD groups. Strictly speaking, one cannot exclude the possibility that the increase in apoptosis of structural cells is only related to cigarette smoking per se, rather than being an event that is specifically associated with the development of COPD. The ideal situation would be to compare COPD patients with heavy smokers who did not develop COPD. Another confounding factor could be the difference in treatment between patients. Most of the studies discussed above do not discriminate between COPD patients that are treated with inhaled corticosteroids and those who are not. It has been demonstrated that corticosteroids induce apoptosis of airway epithelial cells and eosinophils in asthma. [32]. No such data are available for COPD, but these findings underscore the importance of taking into account the use of inhaled steroids when examining apoptosis in the airways of COPD patients.

Thirdly, there are important differences between the different studies regarding the patient population: some groups identified COPD patients by pulmonary function tests, while others studied mainly emphysema patients, as defined by the use of radiologic or histological data. From these studies, it is unclear whether apoptosis is an underlying disease mechanism only for the development of emphysema, or on the contrary, if it is also involved in the disease process of COPD patients without emphysema (and with predominantly bronchiolitis).

Finally, while most groups studied apoptosis of structural cells, it would be interesting to evaluate changes in apoptosis of inflammatory cells in the lungs of COPD patients as well. It has been suggested that chronic inflammation in the airways might result from reduced apoptosis of inflammatory cells, with accumulation of inflammatory cells and sustained inflammation as a consequence [33].

#### Apoptosis outside the lungs

COPD is currently regarded as a multi-component disease with systemic manifestations in addition to local pulmonary inflammation [34]. The lung is of course the principal organ affected by the disease, but the pulmonary manifestations of the disease are often accompanied by systemic abnormalities. This seems to be the case for the disturbance of the balance between apoptosis and regeneration too: while apoptosis of structural lung cells has been demonstrated in COPD patients, several groups described alterations in apoptosis or apoptotic signals in the systemic circulation or in skeletal muscles from COPD patients.

An increased propensity of peripheral blood T cells in COPD to undergo apoptosis has been described [35]. This was accompanied by upregulation of several mediators involved in the induction of T cell apoptosis, such as TNF-α/TNFR1, Fas and TGFR. The authors hypothesized that increased rates of T cell apoptosis result in unbalanced homeostasis, defective clearance mechanisms and perpetuation of the inflammatory response.

Takabatake and colleagues described significantly higher TNF-α and sTNF-R55 and R75 levels in the circulation of COPD patients, while serum levels of soluble Fas ligand (sFas-L), an inducer of apoptosis, and plasma levels of the soluble Fas receptor (sFas), an inhibitor of apoptosis, were not increased in COPD patients. [36].

Others described a significant increase in sFas in plasma from severe COPD patients compared to patients with mild or moderate COPD, while sFas-L was within normal limits in all groups. [37].

Peripheral muscle weakness, due to muscle atrophy, is commonly observed in COPD patients. [38,39]. A possible mechanism of this muscle wasting could be a decrease in the number of muscle fibres resulting from activation of apoptotic pathways. It has been reported that skeletal muscle apoptosis is increased in patients with COPD having a low body mass index (BMI) compared to COPD patients with normal BMI and to healthy volunteers and is associated with a lower exercise capacity [40]. Osteoporosis is another systemic manifestation of COPD [41]. The precise mechanisms involved are unknown and it is unclear if apoptosis contributes to the development of osteoporosis in COPD patients. In summary, a limited number of studies investigated changes in apoptosis outside the lung in COPD patients. The relevance of these findings in the development of COPD is unknown. Future studies will need to investigate in more detail the relation between apoptosis in- and outside the lung in COPD and the importance of apoptosis in the development of systemic manifestations in the course of the disease.

### Animal models of COPD and emphysema

When interpreting data obtained from animal models of COPD, it is important to keep in mind that the development of emphysematous lesions in the lungs of cigarette smoke exposed mice is strain dependent. [42,43]. DBA/2 mice, for example, develop patchy emphysema in response to cigarette smoke faster than C57Bl/6J mice. Additionally, in DBA/2 mice, the development of emphysema is preceded by the appearance of apoptotic cells in areas with a low signal for VEGF-R2 [43], while this is much less the case for C57Bl/6J mice.

Moreover, animal models of COPD often do not represent all characteristics of the disease as it occurs in humans. The current view on the pathogenesis of COPD is that cigarette smoke induces the recruitment of inflammatory cells, which then release reactive oxygen species and proteolytic enzymes, causing the degradation of lung matrix and the death of structural cells. However, in several animal models of COPD, development of emphysema was observed despite a remarkable lack of pulmonary inflammation. These studies demonstrated that, at least in animal models, apoptosis of alveolar wall or endothelial cells is sufficient to cause pulmonary emphysema, even without the accumulation of inflammatory cells. In those studies, emphysema was induced by directly targeting the alveolar cells [44], or by inactivating VEGFR [45-47] or VEGF [48].

The relationship between VEGF, endothelial cell apoptosis and emphysema has first been described by Kasahara and colleagues [45]. These authors demonstrated in a rat model that blocking of the VEGF receptor induced alveolar cell apoptosis and led to enlargement of the airspaces. Moreover, treatment with a caspase inhibitor prevented septal cell apoptosis and emphysema development in response to the blocking of VEGF receptor.

Other groups showed that cathepsin S-dependent epithelial cell apoptosis is a critical event in the pathogenesis of IFN-γ induced emphysema [49]. Using this model, it was recently shown that IFN-γ is a potent activator of the extrinsic/death receptor and intrinsic/mitochondrial apoptosis pathways and that these activation events are partially CCR5 dependent [50].

Aoshiba and colleagues demonstrated that intratracheal administration of active caspase-3 resulted in epithelial apoptosis, enhanced elastolytic activity in BAL and the development of emphysematous changes in mice [44].

Recently, Petrache et al reported that intratracheal instillation of ceramide, a highly regulated sphingolipid second messenger, triggers apoptosis of alveolar epithelial and endothelial cells and induced airspace enlargement in mice [47]. Moreover, increased lung ceramide levels were detected in the lungs of emphysema patients, suggesting that ceramide upregulation might be an important pathogenetic element in the development of emphysema. This was the first study to describe the involvement of a non-protein mediator of apoptosis in the pathogenesis of emphysema.

Others have suggested that humoral- and CD4+ cell- dependent mechanisms can lead to alveolar septal cell apoptosis and the development of emphysema [51]. Intraperitoneal injection of endothelial cells led to the production of antibodies against endothelial cells, influx of CD4+ T cells in the lung, alveolar septal apoptosis, activation of matrix metalloproteinases and the development of emphysema.

### Apoptosis and interaction with other pathogenetic mechanisms in COPD

**Figure 3:**
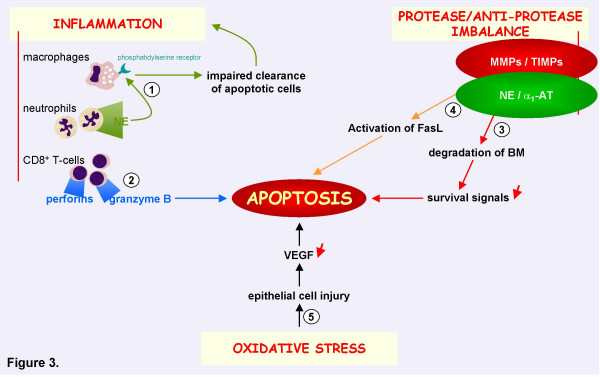
**Interaction of apoptosis with other pathogenetic mechanisms in COPD, including inflammation, oxidative stress and protease/anti-proteinase imbalance**. 1. Neutrophil elastase (NE) cleaves the phosphatidylserine receptor on macrophages, resulting in impaired clearance of apoptotic cells and sustained inflammation [54]. 2. Cytotoxic CD8+ T-cells cause apoptosis of alveolar epithelial cells through the release of perforins and granzyme-B [57,58]. 3. Degradation of the basement membrane (BM) by matrix metalloproteinases (MMPs) leads to loss of survival signals and induces apoptosis of epithelial cells [60]. 4. Apoptosis may also be affected by direct proteolysis of death-inducing signals. It has been shown that MMP-7 sheds and activates Fas ligand (FasL) that is produced by epithelial cells, thereby mediating apoptosis [62]. 5. Oxidative stress could lead to a reduction of Vascular Endothelial Growth Factor (VEGF) levels, resulting in apoptosis of alveolar endothelial cells [29]. NE: neutrophil elastase; BM: basement membrane; MMPs: matrix metalloproteinases; TIMP: tissue inhibitor of metalloproteinase; α1-AT: α1-anti-trypsin; FasL: Fas ligand; VEGF: vascular endothelial growth factor.

As mentioned before, several disease mechanisms are involved in the development of COPD: inflammation, proteinase/anti-proteinase imbalance and oxidative stress. Apoptosis interacts with all of these pathways, adding to the complexity of the disease (Figure 3).

#### Apoptosis and inflammation

Several groups demonstrated that, at least in animal models, apoptosis of alveolar wall or endothelial cells is sufficient to cause pulmonary emphysema, even without the accumulation of inflammatory cells. However, in the lungs of COPD patients, there is an impressive influx of inflammatory cells and interactions between inflammatory and apoptotic mechanisms most probably take place.

Alveolar macrophages from patients with COPD are less effective in phagocytosing apoptotic airway epithelial cells compared to controls [51]. This might be mediated by the presence of activated numbers of neutrophils in COPD. [52,53]. It has been shown that neutrophil elastase cleaves the phosphatidylserine receptor on macrophages, resulting in impaired clearance of apoptotic cells and sustained inflammation [54].

In addition to neutrophils and macrophages, there is also an increase in CD8+ T-lymphocytes in the lungs of COPD patients. [55,56]. These cytotoxic CD8+ T-cells could cause apoptosis of alveolar epithelial cells through the release of perforins, granzyme-B and TNF-α. [57,58].

#### Apoptosis and proteinase-antiproteinase imbalance

The increase in proteolytic activity in the lungs of COPD patients might interfere with apoptosis in several ways. [59]. The basal membrane contains signals for cell survival and loss of these survival signals (as a consequence of degradation of the basement membrane by matrix metalloproteinases) can induce apoptosis. This process of apoptosis induced by loss of appropriate cell-matrix contacts (called anoikis) is involved in tissue homeostasis by maintaining the correct cell number of high turnover epithelial tissues. [60]. Aoshiba and colleagues already suggested that cell-extracellular matrix interactions modulate apoptosis in bronchial epithelium. [61]. Moreover, they recently reported elastolytic activity in apoptotic lung epithelial cells in a mouse model of emphysema [44]. Apoptosis may also be affected by direct proteolysis of death-inducing signals. It has been shown that MMP-7 sheds and activates Fas ligand that is produced by epithelial cells, thereby mediating apoptosis [62]. Finally, recent data suggest that MMP-8 has anti-inflammatory effects on airway inflammation due to a regulation of inflammatory cell apoptosis [63].

#### Apoptosis and oxidative stress

In a rat model of emphysema induced by VEGFR blockade, Tuder et al demonstrated that apoptosis predominated in the lung in areas of oxidative stress and that experimental blockade of apoptosis markedly reduced the expression of markers of oxidative stress [46]. The administration of a compound with antioxidant activity prevented the development of alveolar cell apoptosis and airspace enlargement, suggesting a positive feedback interaction between oxidative stress and apoptosis. Other groups have shown that mice with impaired expression of antioxidant genes have increased numbers of apoptotic alveolar septal cells (predominantly endothelial and type II epithelial cells) and develop early and extensive emphysema in response to cigarette smoke. [64]. Recent work from Kanazawa and colleagues nicely demonstrated elevated oxidative stress levels and a reciprocal reduction of VEGF levels in induced sputum from COPD patients. [29]. These changes increased with severity of the disease. These findings confirm the relationship between oxidant-antioxidant imbalance and VEGF-dependent homeostasis of alveolar walls in the lungs of COPD patients. The authors hypothesize that epithelial cell injury mediated by oxidative stress induces a decrease in lung VEGF levels, resulting in the development of COPD. These data clearly point out that apoptosis is not an isolated event in the development of COPD and that it interferes with other underlying disease mechanisms.

## Conclusion

An increasing number of data, both from animal models and studies on human subjects, supports an important role for apoptosis in the pathogenesis of COPD.

More studies are needed to identify the most important apoptotic pathways and the caspases involved in the development of COPD. Moreover, it has to be evaluated if apoptosis can be used as a therapeutic target to prevent further deterioration of the disease, which occurs even after the patients have quit smoking. Finally, as disturbance of the balance between apoptosis and regeneration of structural lung cells seems important in the destruction of healthy lung tissue, it would be interesting to evaluate the potential use of stem cell therapy for emphysema.

## Abbreviations

CCR5: chemokine receptor 5

COPD: Chronic Obstructive Pulmonary Disease

IFN-γ: interferon-γ

MMP: Matrixmetalloproteinase

NE: Neutrophil Elastase

SFas: soluble Fas receptor

sFas-L: soluble Fas ligand

STNF-R: Soluble TNF receptor

TGFR: Transforming Growth Factor Receptor

TIMP: Tissue Inhibitor of Metalloproteinases

TNF: Tumour Necrosis Factor

TNFR: Tumour Necrosis Factor Receptor

VEGF: Vascular Endothelial Growth Factor

VEGF R2: Vascular Endothelial Growth Factor Receptor 2

WHO: World Health Organisation

## Competing interests

The author(s) declare that they have no competing interests.
